# Protective RNA nanovaccines against *Mycobacterium avium* subspecies *hominissuis*

**DOI:** 10.3389/fimmu.2023.1188754

**Published:** 2023-06-08

**Authors:** Bubacarr J.B. Touray, Mostafa Hanafy, Yashdeep Phanse, Rachel Hildebrand, Adel M. Talaat

**Affiliations:** ^1^ Department of Pathobiological Sciences, School of Veterinary Medicine, University of Wisconsin, Madison, WI, United States; ^2^ Department of Microbiology and Immunology, Faculty of Veterinary Medicine, Cairo University, Giza, Egypt; ^3^ Pan Genome Systems, Madison, WI, United States; ^4^ Vireo Vaccines International, LLC, Madison, Wisconsin, United States

**Keywords:** mRNA-vaccines, mycobacteria, nanoadjuvants, immunology, NTM = nontuberculous mycobacteria

## Abstract

The induction of an effective immune response is critical for the success of mRNA-based therapeutics. Here, we developed a nanoadjuvant system compromised of Quil-A and DOTAP (dioleoyl 3 trimethylammonium propane), hence named QTAP, for the efficient delivery of mRNA vaccine constructs into cells. Electron microscopy indicated that the complexation of mRNA with QTAP forms nanoparticles with an average size of 75 nm and which have ~90% encapsulation efficiency. The incorporation of pseudouridine-modified mRNA resulted in higher transfection efficiency and protein translation with low cytotoxicity than unmodified mRNA. When QTAP-mRNA or QTAP alone transfected macrophages, pro-inflammatory pathways (e.g., NLRP3, NF-kb, and MyD88) were upregulated, an indication of macrophage activation. In C57Bl/6 mice, QTAP nanovaccines encoding Ag85B and Hsp70 transcripts (QTAP-85B+H70) were able to elicit robust IgG antibody and IFN- ɣ, TNF-α, IL-2, and IL-17 cytokines responses. Following aerosol challenge with a clinical isolate of *M. avium ss. hominissuis (M.ah), a* significant reduction of mycobacterial counts was observed in lungs and spleens of only immunized animals at both 4- and 8-weeks post-challenge. As expected, reduced levels of *M. ah* were associated with diminished histological lesions and robust cell-mediated immunity. Interestingly, polyfunctional T-cells expressing IFN- ɣ, IL-2, and TNF- α were detected at 8 but not 4 weeks post-challenge. Overall, our analysis indicated that QTAP is a highly efficient transfection agent and could improve the immunogenicity of mRNA vaccines against pulmonary *M. ah*, an infection of significant public health importance, especially to the elderly and to those who are immune compromised.

## 1 Introduction

Nucleic acid (NA) vaccines have demonstrated improved safety and tolerability compared to traditional vaccines (1–4), especially with the worldwide use of an mRNA vaccine to control the SARS-CoV-2 pandemic. Unlike live-attenuated vaccines (LAV), NA vaccines encode individual or a myriad of immunogenic antigens to trigger protection without the untoward effect of other factors encoded by the LAV. However, NA vaccines are often less immunogenic than LAV and therefore need to be formulated with an adjuvant to boost their immunogenicity (5–7). Over the past few decades, progress has been made toward identifying novel adjuvants to boost the immune response generated by NA vaccines against both infectious and non-infectious diseases (5, 8–11) including a class of lipid nanoparticles (LNPs) to help with the delivery of vaccine antigens. LNPs have been used as both adjuvants and delivery vehicles for mRNA vaccine constructs (12–14). The LNPs protect mRNA from host endonucleases and promote efficient cellular uptake of constructs for efficient protein translation in cells (15–17). LNPs made from cationic lipids such as 1,2-di-O-octadecenyl-3-trimethylammonium-propane (DOTMA) and its biodegradable analog DOTAP are part of mRNA-based vaccine formulations against several cancers and autoimmune encephalomyelitis (18, 19). Recently, LNP adjuvants were used in two mRNA-based vaccines created by BioNTech/Pfizer and Moderna that targets SARS-Cov-2 virus and have shown protective efficacy in both animal and human studies (20, 21). In this report, we developed a nanoadjuvant system of both Quil-A and DOTAP called QTAP. The developed QTAP nanovaccine adjuvant was tested against *M. ah* causing pulmonary infection in immunocompromised patients.

Adjuvant systems are a combination of immune stimulants that enhance the immunogenicity of vaccine antigens. A purified version of Quil-A (QS-21) has been shown to be less toxic in both mice and humans and is part of approved vaccines against malaria (Mosquirix) and shingles (Shingrix) with high immunogenicity and protective efficacy (22, 23). The QS-21 stimulates both antibody-based and cell-mediated immune responses, eliciting a Th-1-biased immune response with the production of high titers of antibodies (IgG2a and IgG2b, in addition to IgG1), as well as antigen-specific cytotoxic T lymphocytes (24). These studies clearly demonstrate the importance of adjuvants in vaccine formulations especially against challenging pathogens. Combining the efficacy of mRNA vaccines, delivery functions of LNPs, and the inflammatory effect of adjuvants may be a suitable approach to enhance the overall efficacy of nucleic acid-based vaccines.

The application of mRNA vaccines against intracellular pathogens for which no effective vaccine has been developed remains unexplored (25). Currently, there is no licensed vaccine for *M. ah* infection, a significant health problem for the aging and immunocompromised population (26, 27). In pursuit of addressing this problem, several platform technologies were tested before including nucleic acid (**NA**) vaccines (28–30). Mycobacteria rely on a plethora of antigens to drive its virulence in the host making vaccine development a challenge (31, 32), and *M. ah* is not an exception. Previously, RNA as a booster to protein vaccines against *M. ah* showed protective efficacy in mice (33). We suggest that mRNA vaccines targeting a complex pathogen such as *M. ah* would require a mixture of antigens combined with an efficient adjuvant system to provide protective immunity, as suggested before (33). In this report, we describe our efforts towards the development of nanoadjuvant systems with improved physicochemical characteristics and their application to generate protective immunity against aerosol challenges with *M. ah.* To the best of our knowledge, this is the first nanoadjuvant system that combines DOTAP, Quil-A (QTAP), and mRNA at a ratio that ensures efficient cellular uptake, mRNA delivery, protein translation, enhanced immune activation and elicits protective immunity in a murine challenge model. Not only QTAP-based constructs were more stable for prolonged times at different storage temperatures, but also, they were able to efficiently transfect immune cells and induce their activation without antigens. Moreover, when antigens were added, the QTAP adjuvanted mRNA (QTAP-Ag85B+Hsp70) increased macrophage activation and generated localized immunity in immunized mice with the presence of polyfunctional T-cells and reduced tissue colonization with the challenge strain of *M. ah*. Together, these results provide clear evidence of the novel QTAP as a transfection and vaccine nanoadjuvant system that can efficiently deliver mRNA constructs and elicit a protective immune response against *M. ah* infection and potentially other intracellular pathogens.

## 2 Materials and methods

### 2.1 Bacterial cultures and plasmids

For all challenge studies, a *Mycobacterium avium* subspecies *hominissuis* clinical isolate from the collection of the Wisconsin State Laboratory of Hygiene (designated *M. ah* W14 or *M. ah*) was grown and its genome sequenced as detailed before (34). For culturing, *M. ah* W14 was grown in Middlebrook 7H9 broth (BD Biosciences, Sparks, MD, USA) supplemented with 10% DC (2% glucose, 5%, and 0.85% NaCl) in a shaking incubator at 37°C. Bacterial cultures were harvested and stored as before (35). Sequences for mycobacterial genes (*hsp70, Ag85B)*, green fluorescent protein (GFP) from *jellyfish Aequorea Victoria, and* Luciferase (Luc) gene from firefly luciferase were downloaded from GenBank and amplified followed by cloning onto an expression vector pCMV from our laboratory collection, as described before (36). These vectors were used as templates for *in vitro* RNA synthesis using HiScribe^®^ T7 ARCA mRNA Kit (with tailing) (NEB, Ipswich, MA, USA).

### 2.2 Animal vaccinations

To examine nanovaccine safety and immunogenicity, preparations of QTAP-mRNA encoding mycobacterial antigens (Ag85B and Hsp70) were evaluated in 3-weeks old C57BL/6 mice. Mice were purchased from Jackson Laboratory (Bar Harbor, ME, USA) and randomly divided into 3 groups (N=14/group), inoculated through the subcutaneous route with 3 doses (15 μg each) of QTAP-mRNA encoding different antigens (Ag85B+Hsp70) at 5-weeks intervals while other groups were inoculated with PBS or 15 μg of QTAP alone to serve as controls. Mice were monitored for general distress, depression, or inappetence and weight changes over the course of the 15 weeks. At each vaccination time-point, blood samples were collected, and serum was separated for cytokine analysis. For some groups used to examine vaccine protective immunity, mice (N=3) were euthanized from each group at 5 weeks after final immunization to harvest lung and spleen for flow cytometry and histopathology. The remaining 12 mice in each group were infected with 100 CFUs of *M. ah* through the aerosol route. After 48 h, mice (N=2) were euthanized from each group, and their lungs and spleen were harvested and homogenized for infection dose determination by CFU count. At 4 and 8 weeks post-challenge, mice (N=5) were euthanized from each group and their organs (lung, spleen, liver) were harvested and homogenized for flow cytometry, histopathology, and CFU enumeration as detailed before (36, 37).

### 2.3 Preparation and characterization of QTAP nanovaccines

Modified mRNA was synthesized using the HiScribe™ T7 ARCA mRNA Kit (with tailing) (New England BioLabs (NEB) E2060S) and Pseudouridine-5’-Triphosphate - (TriLink N-1019) (San Diego, CA, USA). Briefly, capped modified mRNAs were synthesized by co-transcriptional incorporation of Anti-Reverse Cap Analog (ARCA, NEB #S1411) using T7 RNA Polymerase in the presence of 10 mM Pseudo-UTP. This is followed by DNase I treatment to remove template DNA, and treatment with poly (A) polymerase for poly (A) tail addition. The resulting mRNA is purified by column purification, quantified by Nanodrop, and quality assessed using gel electrophoresis.

To prepare QTAP-mRNA, DOTAP (18:1 TAP (DOTAP) 890890) was purchased from Avanti Polar Lipids (Birmingham, AL, USA) without purification and dissolved in 2% glucose water to a final concentration of 10%. The Quil-A (VET-SAP, Desert King) stock solution of 0.2% was made in nuclease-free water. For each preparation, mRNA, Quil-A, DOTAP, and the buffers were combined at a nitrogen to phosphate (NP) ratio of 4.05 to form QTAP-mRNA. Size distribution and zeta potential of QTAP-mRNA in aqueous dispersion were measured by dynamic light scattering (DLS) on a Malvern Zetasizer instrument at 25°C. For zeta potential measurement, an aliquot (5  μl) of QTAP-NPs was diluted in Alpha-q water and placed in a disposable capillary zeta potential cell, available from the Zetasizer Nano series (38). Transmission Electron Microscopy was performed at the Medical School Electron Microscopy Facility of the University of Wisconsin-Madison using a Philips CM120 transmission electron microscope (FEI, Eindhoven, the Netherlands) at 80 kV. For encapsulation efficiency QTAP-NPs loaded with mRNA were resuspended in 600 μl of 0.05 M phosphate-buffered saline (PBS, pH 7.4) at 37°C. At each time point, suspensions were removed and centrifuged at 14,000 relative centrifugal force for 10 min. The supernatant was removed and replaced with PBS and returned to incubation. Supernatant samples were quantified for released mRNA from the QTAP using a NanoDrop spectrophotometer and compared to the total mRNA used (39, 40).

### 2.4 Cell viability and transfection efficiency of QTAP

Cell viability following mRNA transfection was measured using MTT assay (Millipore Sigma 11465007001, Burlington, MA, USA). Baby Hamster Kidney (BHK) cells (American Type Culture Collection (ATCC), Manassas, VA) were cultured in 96-well plates and transfected for 24 h at 37°C and 5% CO2. The medium was removed and replaced with 10 μl of the MTT labeling reagent (final concentration 0.5 mg/ml) and incubated for 4 h at 37°C and 5% CO2. A 100 ul of the solubilization solution was added to each well and incubated at 37°C and 5% CO2 overnight. The absorbance was recorded using an ELISA plate reader at wavelength 550. To determine transfection efficiency, BHK cells, HEK293T cells, were cultured in Dulbecco’s modified Eagle’s medium (DMEM) (Gibco 31966-021, Waltham, MA, USA) medium supplemented with 10% FBS (Sigma F7524) and penicillin-streptomycin (Gibco 15140-122) whiles J774.A macrophages were cultured in RPMI 1640 (Corning 10-040-CM) medium supplemented with 10% FBS (Sigma F7524) and penicillin-streptomycin (Gibco 15140-122). Cells were seeded at 300,000 density and incubated at 37°C and 5% CO2 until they reach 70-80% confluency followed by transfection with QTAP-NPs encapsulating mRNA. A commercial transfection reagent TransIT^®^-mRNA Transfection Kit (Mirus 2250, Madison, WI) and in-house made DOTAP-NPs were used to transfect an equal amount of mRNA in separate wells as transfection controls. At 24h, 48h, and 72h post-transfection, media were removed, and cells were washed with PBS. Cells were lifted from the plate by gently pipetting up and down and transferred to a 2 ml centrifuge tube and centrifuged at 1500 g for 5 minutes at 4^0^C. For flow cytometry, cells were run on a BD LSR Fortessa flow cytometer. Data were analyzed with FlowJo software (BD Bioscience). The strategy for gating on GFP+ cells was debris exclusion on the forward scatter (FSC) vs side scatter (SSC) dot plot, followed by exclusion of dead cells by fixability dye eFluor 780 (number 65-0865-14; Invitrogen) staining. From the live cell population, total GFP+ cells were gated. Finally, the mean fluorescence intensity of the GFP+ population was determined.

### 2.5 Western blot analysis

Cells were transfected with mRNA as described above. After 24h of incubation, cells were detached and washed with ice-cold PBS, lysed with 1% (w/v) SDS, followed by sonication using Misonix Ultrasonic Liquid Processor sonicator 3000. The total protein for each sample was separated by SDS-PAGE, and transferred to a poly(vinylidene difluoride) membrane; proteins were detected by western analysis with the histidine-tag antibodies ad detailed before (41).

### 2.6 Flow cytometric assessment of QTAP-mRNA immunogenicity and protective efficacy

Lungs and spleens collected from vaccinated mice were used for flow cytometric assessment. Briefly, tissues were excised and placed in a gentleMACS dissociator M Tube (Miltenyi 130-093-236, Bergisch Gladbach, Germany) with 3 mL collagenase B (1 mg/mL) (Roche, Basel, Switzerland) and incubated for 30 min at 37°C. Single-cell were prepared by gently squeezing through a 70-mm cell strainer (Falcon) after lysing RBCs using 1X BD Biosciences BD Pharm Lyse™ (San Jose, CA, USA). For intracellular cytokine staining, 10^6^ cells were stimulated with *M. ah* purified protein derivative (PPD) (1 mg/ml) and IL-2 (400U/ml) while an equal concentration of IL-2 was added to the remaining replicate as unstimulated control. After 16h incubation at 37°C, 5% CO_2_, Brefeldin A (1 μL/mL, GolgiPlug, BD Biosciences) was added, and the cells were further incubated for an additional 6 h at 37°C, 37°C, 5% CO_2_. Fluorochrome-labeled antibodies against the cell-surface antigens CD4 (BUV 496, GK1.5), CD8a (BUV395, 53-6.7), and intracellular antigens IFN-γ (APC, XMG1.2); TNF-α (BV421, MP6-XT22); IL-2 (PE-CF594, JES6-5H4); IL-17 (FITC, TC11-18H10.1) were purchased from BD Biosciences; Biolegend (San Diego, CA, USA); eBioscience (San Diego, CA, USA); or Invitrogen (Grand Island, NY, USA). Before antibody staining, the cells were stained for viability with Dye eFluor 780 (eBiosciences, San Diego, CA, USA). After stimulation, the cells were stained for surface markers and then processed with the Cytofix/Cytoperm kit (BD Biosciences, San Jose, CA, USA). To stain for cytokines, the cells were first stained for cell-surface molecules, fixed, permeabilized, and subsequently stained for the cytokines. All samples were acquired on an LSR Fortessa flow cytometer (BD Biosciences, San Jose, CA, USA). Data were analyzed with FlowJo software (TreeStar, Woodburn, OR, USA). Results are expressed as the difference in the percentage of stimulated cells with that of unstimulated cells. At least 100,000 events were collected for each sample. A Boolean gating strategy was applied for the determination of cytokine-secreting T cells.

### 2.7 ELISA assay

Serum samples were collected from animals at designated times and stored at -80°C until use. After thawing, sera were 1:10 diluted with buffer (PBS-Tween 0.05% with 1% BSA) to obtain a working concentration for the ELISA. ELISA plates (96-well) were coated with *M. avium* purified protein derivatives (PPD) at a concentration of 10 µg/mL in carbonate-bicarbonate buffer (pH 9.6). The plates were incubated overnight at 4°C, washed with PBS-Tween 0.05%, and blocked with 200 µL of blocking buffer (PBS-Tween 0.05% with 3% BSA) for 1 hour at room temperature. After blocking, plates were washed and 100 µL of the diluted serum samples were added to each well for 2 hours at room temperature. After incubation, the plates were washed and 100 µL of HRP-conjugated anti-mouse IgG antibody (diluted 1:5000 in sample diluent buffer) was added to each well. The plates were then incubated for 1 hour at room temperature, washed and 100 µL of TMB substrate solution was added to each well. After 10-15 minutes of incubation at room temperature, protected from light, color development was stopped by adding 50 µL of 2M sulfuric acid to each well. Plates were read at 450 nm using a microplate reader.

### 2.8 Statistical analysis

Statistical analyses were performed using GraphPad software (La Jolla, CA, USA). Nanoparticle size, protein expression, thermostability, and cytotoxicity were compared using ordinary one-way ANOVA where *p < 0.05, **p < 0.01, ***p < 0.001, and ****p < 0.0001 were considered significantly different among groups. Serum cytokine and cellular immune assays were compared using an ordinary one-way ANOVA test where *, *p* < 0.05; **, *p* < 0.01 were considered significantly different among groups.

## 3 Results

### 3.1 Characterization of QTAP-mRNA nanovaccine

**Figure 1:**
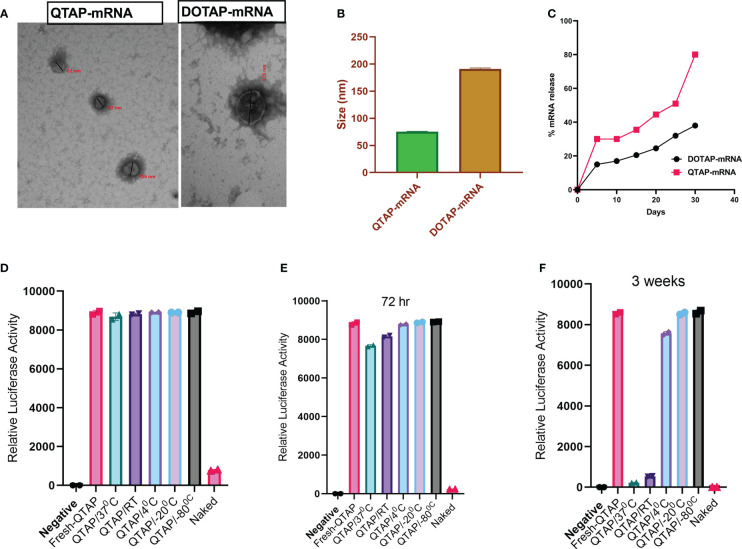
QTAP forms nanoparticles and releases RNA cargo in a sustained manner with high stability. **(A)** Electron microgram of QTAP and DOTAP-encapsulated mRNA measured by TEM. **(B)** DLS data of NPs measured at 25°C with Zetasizer software. **(C)** *In vitro* sustained release kinetics of packaged mRNA measured at pH-7.4, 37°C. **(D–F)** Relative expression of Luc protein in BHK cells transfected with QTAP-encapsulated Luc mRNA stored at different temperatures measured by luminometry. Data were plotted in GraphPad Prism and *one-way ANOVA was* used to examine differences between samples.

To facilitate the characterization of QTAP-mRNA nanovaccines, we first formulated modified mRNA-encoding reporter genes such as luciferase (Luc) or GFP proteins using QTAP encapsulation. The mRNA was modified with the substitution of at least 66% of the native uridine nucleotides to Pseudouridines (Ψ) as suggested before (42, 43). The Ψ-mRNA integrity and purity were assessed by gel electrophoresis and formulated into the Quil-A adjuvanted DOTAP LNPs (QTAP). Transmission electron microscopy (TEM) analysis of QTAP-mRNA encoding GFP showed a few particles (~95 nm in size) which are spherical with no observed particle aggregation **(** **Figure 1A** **)**. Also, dynamic light scattering (DLS) of the QTAP-mRNA complex displayed an average particle size of ~75 nm with a zeta potential of 34 **(** **Figure 1B** **)**. The encapsulation efficiency (EE%) of QTAP nanoparticles (NP) was > 90%. The release kinetics of mRNA from QTAP NPs showed sustained release of up to 80% of the mRNA cargo within the first 30 days of testing **(** **Figure 1C** **).**

To test the stability of QTAP, we encapsulated Ψ-mRNA encoding Luc protein in QTAP and incubated the complex at different temperatures. At all temperatures, QTAP-mRNA is stable for up to 72h with no significant difference to freshly prepared QTAP-mRNA **(** **Figures 1D, E** **)**. Interestingly, after 3 weeks, QTAP-mRNA remains stable at 4 ^0^C, -20 ^0^C, and -80 ^0^C whereas at 37 ^0^C and RT, the stability was reduced significantly after 72h **(** **Figure 1F** **)**. The luciferase activity suggests that QTAP-mRNA forms nanostructures that are stable at higher temperatures and can deliver the mRNA cargo in a sustained release manner.

### 3.2 The combination of DOTAP and Quil-A enhances the delivery of functional modified mRNA in cells

**Figure 2:**
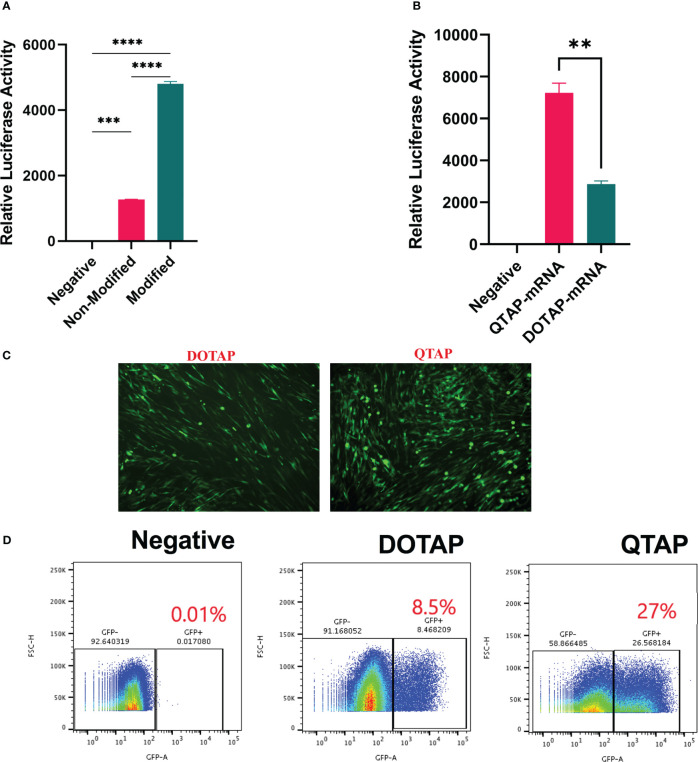
QTAP nanovaccine can efficiently deliver modified mRNA leading to higher protein expression in cells. **(A, B)** Relative expression of Luc protein in BHK cells transfected with QTAP-encapsulated Luc mRNA determined by luminometry. **(C)** Fluorescence microscope image of GFP expression in BHK cells transfected with either DOTAP or QTAP-encapsulated GFP mRNA. **(D)** Transfection efficiency of DOTAP and QTAP-encapsulated GFP mRNA in BHK cells measured by flow cytometry. Data were analyzed and plotted in GraphPad Prism and statistical differences calculated by One-way ANOVA. Asterisks indicate statistical significance, where *p < 0.05, **p < 0.01, ***p < 0.001, and ****p < 0.0001.

To determine the ability of QTAP to efficiently deliver functional Ψ -mRNA, BHK cells were transfected with Ψ-mRNA encoding either Luc or GFP proteins. At 24h post-transfection, Ψ modified mRNA resulted in a 4-fold increase in luciferase expression **(** **Figure 2A** **)**. Similarly, luciferase activity was higher in QTAP-mRNA compared to DOTAP-mRNA transfected cells **(** **Figure 2B** **)**. Cells transfected with QTAP encapsulating Ψ-mRNA encoding GFP showed a higher number of cells expressing GFP than unmodified constructs at 48h (data not shown) suggesting that enhanced protein expression is not dependent on QTAP encapsulation but Ψ presence. Moreover, complexation of Ψ-mRNA encoding GFP with the neoadjuvant (QTAP) showed a significant increase in the number of GFP-expressing cells compared to DOTAP control at 72 h **(** **Figure 2C** **)**. When flow cytometry was used, the number of cells expressing GFP was higher in QTAP-mRNA transfected cells than in DOTAP-mRNA transfected cells **(** **Figure 2D** **)**. Overall, these findings illustrate the ability of the novel QTAP delivery platform to transfect mRNA efficiently in cells leading to detectable protein expression *in vitro*.

### 3.3 QTAP NPs encapsulating modified mRNA of mycobacterial Ag85B activate macrophages and are not cytotoxic

**Figure 3:**
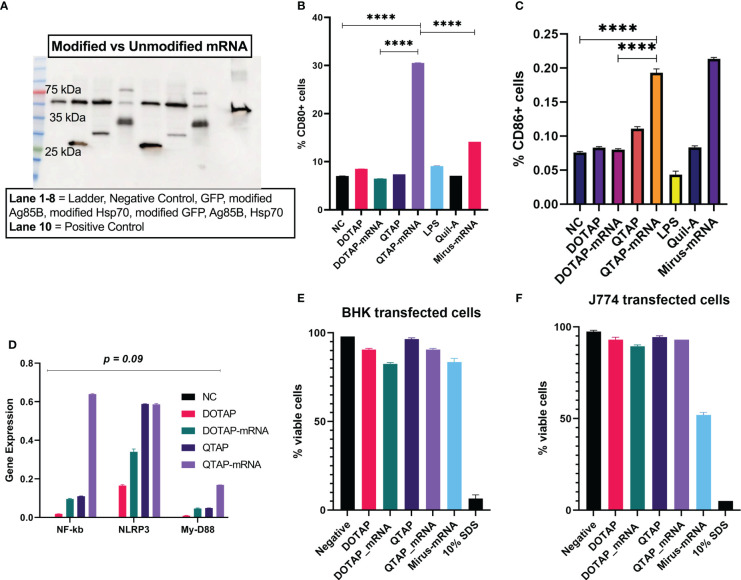
QTAP encapsulating mRNA-Ag85B is not cytotoxic and activates macrophages. **(A)** Protein expression of modified vs non-modified mRNA determined by Western blot analysis using anti-histidine antibody lane 1-10: Ladder, negative control, GFP, modified Ag85B, modified Hsp70, modified GFP, unmodified Ag85B, unmodified Hsp70, positive control (SARS-Cov-2 histidine-tagged Nucleocapsid protein)**. (B, C)** Macrophages transfected with QTAP-Ag85B mRNA were used to examine the cellular expression of macrophage activation markers CD80 and CD86 measured by flow cytometry. **(D)** Activation of macrophage inflammatory pathways in response to QTAP-Ag85B mRNA stimulation determined by One-step qRT-PCR using gene-specific primers. **(E, F)** BHK and J774 cells were transfected with QTAP-mRNA-Ag85B (1 ug) for 24 hr after which viability was determined by MTT cytotoxicity assay. All data were analyzed and plotted in GraphPad Prism and statistical differences were calculated by One-way ANOVA. The black line and error bars show mean ± SD. Asterisks indicate statistical significance, where *p < 0.05, **p < 0.01, ***p < 0.001, and ****p < 0.0001.

To examine how a QTAP nanovaccine can modulate cells, macrophages (J744A.1) were transfected with QTAP followed by flow cytometric acquisition. Western blot analysis of *M. ah* antigens Ag85B and Hsp70 showed increased protein expression from BHK cells after transfection with QTAP-mRNA **(** **Figure 3A** **).** Cells expressing CD80 **(** **Figure 3B** **)** and CD86 **(** **Figure 3C** **)** were significantly higher when transfected with QTAP-Ψ-mRNA-Ag85B than DOTAP-Ψ-mRNA-Ag85B. Interestingly, transfection of cells with either QTAP alone or QTAP-Ψ-mRNA-Ag85B resulted in the upregulation of inflammatory pathways in cell culture **(** **Figure 3D** **)** compared to DOTAP controls. Overall, the novel QTAP NAS can activate macrophages toward an inflammatory state. Finally, we also analyzed the cytotoxicity of QTAP-Ψ-mRNA using MTT cytotoxicity assay on two different cell lines (BHK and J774). The viability of both BHK and J774 cells transfected with DOTAP and QTAP encapsulating Ag85B Ψ-mRNA was not significantly affected compared to the negative control **(** **Figures 3E, F** **)**. On the other hand, Mirus mRNA transfection reagent significantly reduced cell viability in both cells at the same time point. Although at this time point we observed higher transfection efficiency in cells transfected with Mirus mRNA transfection reagent encapsulating Ψ-mRNA encoding GFP, it also resulted in lower viability in both cells. Additionally, evaluation of reactive oxygen species (ROS) production in macrophages showed QTAP-mRNA ROS levels comparable to DMSO. However, DOTAP-mRNA has higher ROS levels than both DMSO and QTAP-mRNA **(**data not shown**)**.

### 3.4 QTAP-based mRNA nanovaccine is safe and elicits a robust immune response in mice.

**Figure 4:**
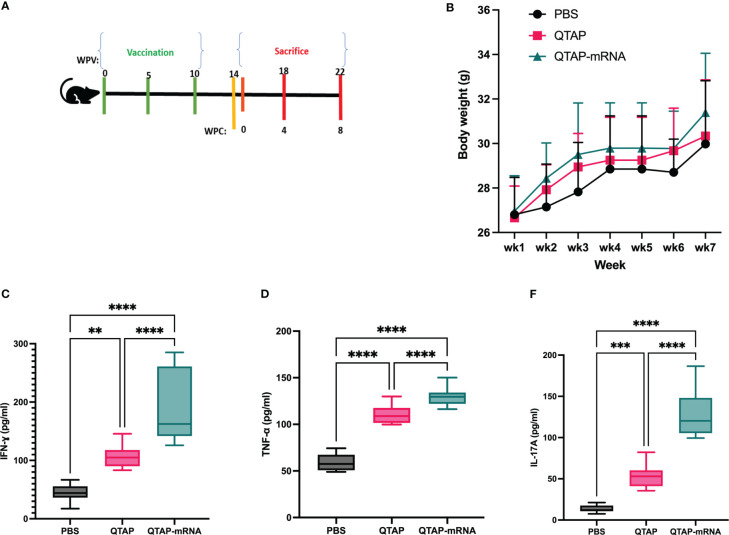
QTAP-mRNA is both safe and immunogenic in mice. **(A)** Immunogenicity of QTAP encapsulating 15 ug of mRNA (Ag85B + Hsp70) was determined in 2-weeks old C57Bl/6 mice using three-dose vaccination at 5 week intervals. **(B)** Mouse weight was measured every week and presented as standard deviation. Serum from vaccinated mice collected after the final vaccine dose and used for cytokine ELISA targeting IFN-γ **(C)**, TNF-α **(D)**, and IL-17 **(E)**. All data were analyzed and plotted in GraphPad Prism and statistical differences were calculated by One-way ANOVA. Dots represent individual mice (n = 10-14/group) and the black line and error bars show mean ± SD. Asterisks indicate statistical significance, where *p < 0.05, **p < 0.01, ***p < 0.001, and ****p < 0.0001.

**Figure 5:**
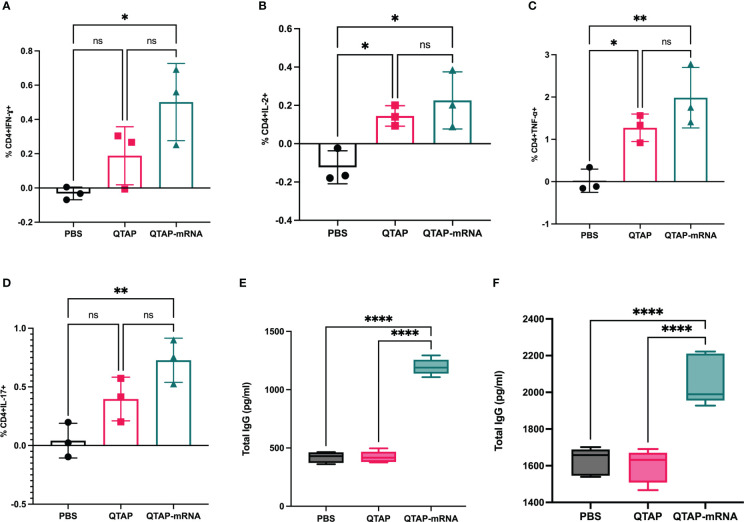
Immunization of mice with QTAP-mRNA Ag85B + Hsp70 is safe and elicits a robust T cell immune response. **(A–D)** Lung cells harvested after the final vaccine dose were stimulated with PPD *ex vivo* and evaluated for CD4 T cell responses by intracellular cytokine staining flow cytometry. Percent frequency of CD4^+^ PPD specific cytokine-producing cells four-weeks post final immunization. Serums collected at 5 weeks after the second vaccine dose **(E)** and four-week post-challenge **(F)** were analyzed for IgG response by ELISA. Data were analyzed by FlowJo and GraphPad prism used for one-way ANOVA analysis. Dots represent individual mice (n = 3/group) and the black line and error bars show mean ± SD. Asterisks indicate statistical significance, where *p < 0.05, **p < 0.01, ***p < 0.001, and ****p < 0.0001.

The safety and immunogenicity of QTAP encapsulating modified mRNA encoding Ag85B and Hsp70 were evaluated in C57Bl/6 mice **(** **Figure 4A** **)**. Mice were monitored for clinical signs such as inflammation at the site of vaccine injection, depression, or inappetence. No clinical signs were observed in all groups over the course of 15 weeks. Although QTAP-mRNA vaccinated mice showed hair loss at the posterior and dorsal regions, we observed no clinical severities in these groups. The weight of mice vaccinated with either QTAP only or QTAP-mRNA groups did not differ from PBS inoculated mice **(** **Figure 4B** **)**. ELISA titers in blood indicated that QTAP-mRNA (Ag85B + Hsp70) elicited significant levels of IFN-γ, TNF-α, and IL-17 specific cytokines compared to PBS control group. Interestingly, the QTAP-only group showed elevated cytokine levels relative to the PBS group suggesting that QTAP by itself is immunogenic **(** **Figures 4C–E** **).** Overall, these findings demonstrate the adjuvant effect of QTAP alone which is amplified by the encapsulated mRNA. Histopathological analysis of the lungs shows no pathological damage (data not shown). At 4 weeks post-final vaccination, we quantified the percentage of CD4+ and CD8+ T cells in the lungs and spleen of mice from all groups and analyzed the population of Th-1 (IFN-γ, TNF-α, IL-2) and Th-17 (IL-17A) cytokine-producing T cells by flow cytometry (**Figure 5** **)**. The results indicate that QTAP and QTAP-mRNA elicit significantly higher proportions of CD 4 T cells secreting pro-inflammatory cytokines in mice compared to the PBS group. However, there were no significant levels of these cytokines in CD8 T cells (data not shown). Interestingly, humoral immune response characterized by the presence of IgG antibodies were detected at significantly higher levels in the sera of QTAP-mRNA immunized mice compared to the control groups. This increase in IgG response was detected as early as 5 weeks after the second vaccine dose and up to at least 4 weeks post-challenge **(** **Figures 5E, F** **)**. Overall, these results suggest that QTAP encapsulating modified mRNA encoding mycobacterial Ag85B and Hsp70 is safe and immunogenic in mice.

### 3.5 Immunization with QTAP nanovaccine protects mice against *Mycobacterium avium ss. hominissuis* infection

**Figure 6:**
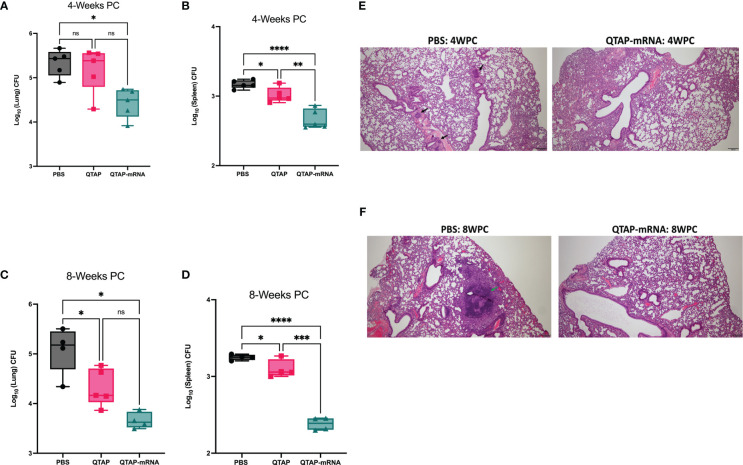
Immunization of mice with QTAP-mRNA Ag85B + Hsp70 is protective against *M. ah* infection for up to 8 weeks. C57BL/6 mice were infected with *M. ah* by aerosol route four weeks post final immunization. **(A, B)** Bacterial burden was determined by colony forming unit (CFU) in the lung and spleen four- and eight-weeks post-challenge **(C, D)**. Dots represent individual mice (n = 4-5/group) and the black line and error bars show mean ± SD. Histological assessment was done by H&E staining of lung tissue sections at 4 weeks **(E)** and 8 weeks **(F)** post-challenge. Arrowheads represent granuloma-like structures. Scale bars represent magnification (5x). Asterisks indicate statistical significance, where *p < 0.05, **p < 0.01, ***p < 0.001, and ****p < 0.0001.

To investigate the protective efficacy of the novel QTAP nanovaccine, we analyzed the bacterial burden and histopathological changes in vaccinated and challenged mice at different times post-challenge (**Figure 4A** **)**. Bacterial counts showed that vaccination of mice with QTAP-mRNA resulted in a significant reduction in bacterial burden in the lungs and spleen at both timepoints **(** **Figures 6A–D** **)** with little pathological damages of the lung airways **(** **Figures 6E, F** **)**. In unvaccinated mice, we observed pre-granuloma structures at 4 weeks post-infection and fully formed granuloma-like structures at 8 weeks post-challenge with extensive damage to the lung airways. Analysis of the spleen showed no noticeable pathological damage in both groups.

**Figure 7:**
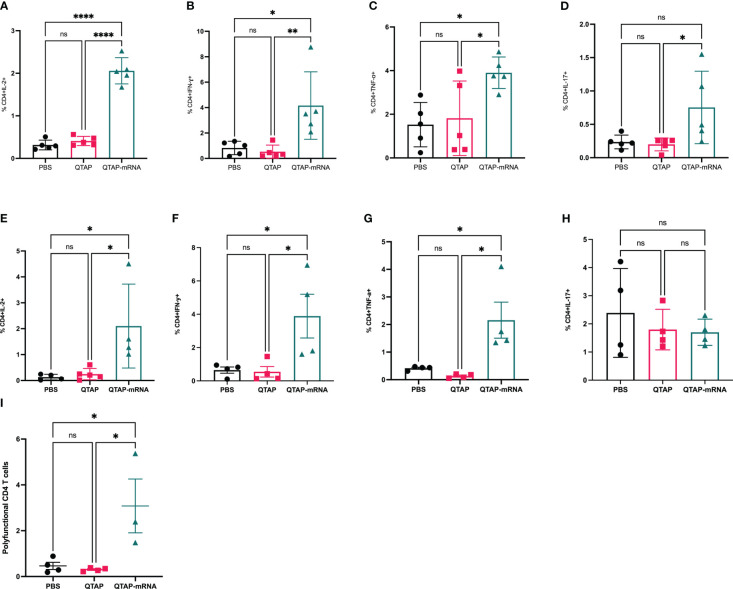
Mice immunized with QTAP-mRNA Ag85B + Hsp70 elicit a robust immune in the lungs upon infection with *M. ah* up to 8 weeks. C57BL/6 mice were infected with *M. ah* by aerosol route four weeks post final immunization. After four- and eight-weeks post-challenge, mice were euthanized for lung and spleen and were evaluated for CD4 T cell responses by intracellular cytokine staining flow cytometry. **(A–D)** Percent frequency of CD4^+^ PPD specific cytokine-producing cells at four weeks post-challenge. **(E–H)** Percent frequency of CD4^+^ PPD specific cytokine-producing cells at eight weeks post-challenge. **(I)** Percent frequency of CD4^+^ PPD specific cytokine-producing IFN-γ, IL-2, and TNF- α cells at eight weeks post-challenge. Data were analyzed by FlowJo and GraphPad prism used for one-way ANOVA analysis. Dots represent individual mice (n = 4-5/group) and the black line and error bars show mean ± SD. Asterisks indicate statistical significance, where *p < 0.05, **p < 0.01, ***p < 0.001, and ****p < 0.0001.

To characterize the immune response of protection, vaccinated mice challenged with *M. ah* resulted in a higher number of CD4+ T-cells secreting IFN-γ, IL-2, TNF- α, and IL-17A cytokines compared to PBS and QTAP-only groups at 4 weeks post-challenge (**Figures 7A–D** **)**. At 8 weeks post-challenge, CD4+ and CD8+ (data not shown) T-cells secreting IFN-γ, TNF-α, and IL-2, remain significantly higher in the vaccinated group with no apparent difference in IL-17A among the groups (**Figures 7E–H** **)**. A similar profile for CD8+ T cell response was detected at this timepoint (data not shown). Interestingly, QTAP-mRNA immunized mice had poly-functional CD4 T cells secreting IFN-γ, IL-2, and TNF- α in only their lungs at 8 weeks post-challenge (**Figure 7I** **)**. These findings suggest that the novel QTAP-mRNA nanovaccine is protective against *M. ah* infection in mice.

## 4 Discussion

Recently, mRNA vaccines have attracted great attention since their successful use to combat the COVID-19 pandemic (44, 45). Despite this success, it remains unknown whether the same approach can be used to target more challenging pathogens such as mycobacteria. Unlike SARS-CoV-2, mycobacterial pathogens use complex pathogenic mechanisms consisting of a plethora of virulence factors for evasion of host immune pathways that enable them to either cause disease or remain persistent in the lungs (46, 47). This presents a challenge for designing effective vaccines against mycobacteria. However, the mRNA vaccine technology allows for the careful design of vaccine constructs with the flexibility to include different antigens to target multiple pathogenic pathways (44, 48). We hypothesize that vaccines targeting mycobacterial pathogens should be rationally designed to include multiple antigens to match the complexity of the intracellular life cycle of the bacteria. Indeed, it has been previously shown that RNA vaccine (repRNA-ID91/ID91+GLA-SE) encoding four *M. ah* antigens produced significant cellular and humoral immune responses leading to reduced bacterial burden (33). The same group showed that repRNA-ID91/ID91+GLA-SE provided similar protection against *M. tb* infection in mice (29). However, the highest protection generated by this vaccine was when it is used in a prime (RNA)-boost (protein) regimen (29, 33). Beyond design, mRNA vaccines require sufficient immunostimulatory adjuvants to achieve optimal protective efficacy (49, 50). A recent study has shown that those LNPs made from DOTAP, 1,2-dipalmitoyl-sn-glycero-3-phosphocholine (DPPC), and cholesterol alone failed to provoke inflammatory responses such as pro-inflammatory cytokine production and inflammatory cell infiltration in mice (51). On the other hand, LNPs made with recombinant hemagglutinin (HA) and neuraminidase proteins induce inflammatory responses for influenza seasonal Flu vaccines (51). These findings demonstrate the limitation of LNPs alone in eliciting optimal immune response while also highlighting the importance of additional adjuvants in eliciting pro-inflammatory immune responses which is a pre-requisite for protection against mycobacterial infections (52). The repRNA-ID91/ID91+GLA-SE vaccine-mediated cellular and humoral immune response leading to protective immunity against both *M. ah* and *M. tb* is associated with the GLA-SE adjuvant (29, 33). The primary goal of the studies reported here was to evaluate the novel QTAP nanoadjuvant platform for the development of effective mRNA vaccines targeting *M. ah* infection in mice. The initial studies focused on the mRNA delivery capability of QTAP nanoadjuvant to efficiently entrap and deliver mRNA in cells and elicit an inflammatory profile suitable for the control of mycobacteria in mice.

We show that QTAP nanoadjuvant complexed with mRNA forms nanoparticles (NPs). The physical parameters of QTAP NPs encapsulating mRNA (~75 nm, positively charged) are consistent with previous findings suggesting that positively charged NPs with a size range of 50-150 nm are suitable for induction of sufficient immune response in both mice and non-human primates (53, 54). Under *in vitro* conditions designed to mimic cellular environmental conditions, QTAP NPs released mRNA in a sustained manner over a long period of time. Indeed, previous studies have demonstrated that prolonged release is important for sustained induction of immune response over a long period and enables sufficient lymphocyte activation and proliferation with cytokine induction (55). Additionally, when stored at different temperatures, QTAP NPs protect mRNA from degradation demonstrated by the expression of encoded proteins in cells after transfection (56). Most FDA-approved mRNA vaccines and those in clinical trials require extremely low temperatures for storage (57, 58). This limits their application in areas of the world that harbors the greatest burden of infectious diseases with high temperature and limited access to cold storage. We showed that QTAP NPs can protect mRNA at even higher temperatures (4-20 ^0^C) than the reported temperature requirements of current FDA-approved LNP/mRNA vaccines (57, 58).

QTAP nanoadjuvant encapsulating modified mRNA encoding either Luc, GFP, Ag85B, or Hsp70 gene showed higher protein expression and transfection efficiency compared to DOTAP-mRNA. In BHK cells modified mRNA delivered by QTAP NPs showed higher protein expression similar to previous reports (42, 59). Additionally, we showed that the presence of Quil-A in the LNP/mRNA showed a more than 200% increase in GFP+ cells in QTAP-transfected BHK cells. As previously reported, mRNA-based vaccine immunogenicity and protective efficacy are dependent on the amount of mRNA-derived protein antigens (55). Moreover, we showed that macrophages exposed to QTAP nanoadjuvant encapsulating mRNA-Ag85B exhibit elevated induction of NLRP3 inflammasomes, NF-kb, and My-D88. We observed that QTAP alone did not lead to any increase in CD80 expression in macrophages. However, although statistically insignificant, we noticed that QTAP-treated macrophages have a slight increase in expression of CD86 compared to the negative control. We expected QTAP-mRNA to have higher expression of these co-stimulatory molecules because mRNA by itself is immunogenic. Secondly, QTAP alone showed a slight increase in activation of both NF-kb and My-D88 compared to both negative and DOTAP-only controls. Although statistically insignificant, it is not clear whether such a slight increase in NF-kb and My-D88 activation has any biological significance. LNP-based activation of effector immune cells has been shown to be required for the induction of effective immune response against pathogens (60–62). Due to these reasons, the DOTAP-mRNA group is not included in the animal immunization and challenge experiments since it has minimal transfection efficiency and macrophage activation ability.

Mouse vaccination studies indicated that QTAP-mRNA encoding Ag85B and Hsp70 is safe and highly immunogenic. A dose of 15 ug in a 3-dose immunization regimen, resulted in no significant changes in mouse weight. This immunization regimen elicited robust cell and humoral-mediated immune responses. We observed that immunization of mice with QTAP-mRNA encoding Ag85B and Hsp70 produced both Th-1 and Th-17 immune responses demonstrated by elevated secretion of proinflammatory cytokines IL-2, IFN-γ, TNF-α, and IL-17 by these T cells. Previous studies have shown that the humoral immune response plays a crucial role in the protection of the host against mycobacterial infections. The QTAP-mRNA vaccine elicited a robust antibody response characterized by elevated IgG levels detected in mouse sera very early after immunization and lasting even after infection. Additionally, we showed that the vaccine was not associated with any pathology in the lungs compared to the PBS control group. However, a mild increase in lung infiltrating lymphocytes was seen with no apparent pathological damage of the airway in the vaccinated group. Previously, protein vaccine boosted with RNA against *M. ah* challenge in mice showed reduced lesions in the lung compared to naïve control (33). However, the vaccinated mice had more than 40% of their lungs affected by lesions (33). In the case of QTAP-mRNA, we did not observe such widespread lesions in the lungs even though the vaccine contains only two mycobacterial antigens.

Interestingly, immunized mice challenged with *M. ah* demonstrated significantly reduced bacterial burden in both the lungs and spleen at both 4- and 8-weeks post-challenge. The T cell response profile at these time points was similar to the pre-challenge profile. At 4 weeks post-infection, we observed a significant increase in lung infiltrating IL-2, IFN-γ, TNF-α, and IL-17 secreting CD4 but not CD8 T cells in the vaccinated group. These findings are like the results of previous RNA vaccines against both *M. ah* and *M. tb* in mice (29, 33). In *M. tb* and *M. ah* infections, continuous exposure to the bacteria causes exhaustion in CD8 T cells thereby inhibiting their cytokine secretory capability (63–65). From these findings, in the presence of active CD4 T cell response to *M. ah* in vaccinated mice, the absence of CD8 T cell response did not seem to stop the reduction in infection burden. However, we cannot disqualify the absence of CD8 T cell response as a limiting factor of *M. ah* control. We hypothesize that augmentation of CD8 T cell response will strengthen the protective efficacy of mRNA vaccines against mycobacterial pathogens. Further studies using CD8 T cell depletion will help to decipher the role of CD8 T cells in the QTAP-mRNA nanovaccines mediated protection against *M. ah.* At 8 weeks post-challenge, a similar T cell response was observed in CD4 T cells. However, we noticed that IL-17 was highly elevated in the PBS group unlike at 4 weeks post-challenge, while IL-2, IFN-γ, TNF-α, and IL-17 cytokines maintained similar levels in the vaccinated mice from 4 to 8 weeks. This profile might be due to the Th-1/Th-17 imbalance previously reported in active mycobacterial infections where a shift towards excessive IL-17 response causes extensive neutrophil recruitment and tissue damage (66–69). Indeed, we observed extensive pathological damages only in the lungs of unvaccinated mice with large granuloma structures. Also, unlike the 4-week post-challenge time-point, CD8 T cell responses in the vaccinated mice were enhanced at 8 weeks post-challenge characterized by a significant increase in IL-2, IFN-γ, TNF-α cytokines levels. However, the level of cytokines secreted by CD4 T cells was higher than CD8 T cells. This observation supports previous findings that in the case of mycobacterial infections such as *M. tb*, the primary immune response responsible for protective immunity against infection is CD4 T cell-mediated (70) with evidence of poor CD8 T cell-mediated protection (71). Recent studies have shown that CD8 T cells recognize *M. tb* and have cytolytic functions and produce inflammatory cytokines highlighting their important role in *M. tb* control (72, 73). In other studies, CD4 T cells have been shown to help prevent CD8 T cell exhaustion during *M. tb* infection, and that in the absence of CD4 T cells, CD8 T cell-mediated protection is underestimated (63, 74). These findings demonstrate the importance of both classes of T cells and demonstrate the synergy between CD4 and CD8 T cells in the control of *M. tb* infection. In our case, we showed that the novel QTAP-mRNA vaccine elicits predominantly CD4 T cell response during the early phases of infection whereas the late phase of infection is characterized by both CD4 and CD8 T cell response in vaccinated mice. Additionally, at 8 weeks post-infection, we demonstrated more than 1.5 log reduction of bacterial CFUs in the lungs of the vaccinated mice.

Overall, we show that the use of the novel QTAP nanoadjuvant for the delivery of mRNA vaccine constructs targeting *M.ah* is highly effective in eliciting protective immunity. The QTAP nanoadjuvant is a promising system for the effective delivery of mRNA vaccine constructs for both *in vitro* and *in vivo* models with the added value of thermostability at higher temperatures. While LNP-mRNA vaccines targeting mycobacterial pathogens may prove effective, QTAP nanoadjuvanted mRNA-based vaccines will likely provide long-lasting immunity.

## Data availability statement

The original contributions presented in the study are included in the article/Supplementary Material. Further inquiries can be directed to the corresponding author.

## Ethics statement

The animal study was reviewed and approved by Institutional Animal Care and Use Committee (IACUC) - School of Veterinary Medicine, University of Wisconsin-Madison.

## Author contributions

AT led and developed the conceptual framework of the project with inputs from BT. BT performed all *in vitro* and animal experiments with help from MH and RH. BT analyzed and interpreted the data and wrote the manuscript. AT and YP edited the manuscript. All contributing authors approved the submitted version.
